# Bis(benzyl­trimethyl­ammonium) di-μ-bromido-bis­[dibromido­mercurate(II)]

**DOI:** 10.1107/S1600536811055887

**Published:** 2012-01-07

**Authors:** Lei Jin

**Affiliations:** a College of Chemistry and Chemical Engineering, Southeast University, Nanjing 210096, People’s Republic of China

## Abstract

In the crystal structure of the title compound, (C_10_H_16_N)_2_[Hg_2_Br_6_], the condensed anion consists of two edge-sharing HgBr_4_ tetrahedra and is situated on a centre of symmetry. The anions are linked to the cations through weak C—H⋯Br inter­actions.

## Related literature

For related structures, see: Jin & Liu (2011 ▶); Nockemann & Meyer (2002 ▶).
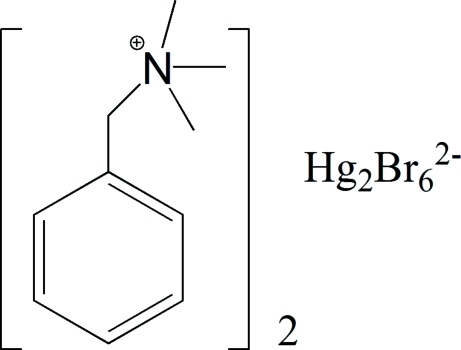


## Experimental

### 

#### Crystal data

(C_10_H_16_N)_2_[Hg_2_Br_6_]
*M* *_r_* = 1181.06
Triclinic, 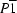

*a* = 9.0542 (11) Å
*b* = 9.7287 (9) Å
*c* = 9.894 (1) Å
α = 80.78 (1)°
β = 71.02 (1)°
γ = 62.39 (1)°
*V* = 730.24 (13) Å^3^
*Z* = 1
Mo *K*α radiation
μ = 18.72 mm^−1^
*T* = 298 K
0.26 × 0.22 × 0.20 mm

#### Data collection

Rigaku Mercury2 diffractometer
Absorption correction: multi-scan (*CrystalClear*; Rigaku, 2005 ▶) *T*_min_ = 0.011, *T*_max_ = 0.024
6877 measured reflections
2872 independent reflections
2166 reflections with *I* > 2σ(*I*)
*R*_int_ = 0.048

#### Refinement

*R*[*F* ^2^ > 2σ(*F* ^2^)] = 0.052
*wR*(*F* ^2^) = 0.131
*S* = 1.07
2862 reflections
139 parameters
H-atom parameters constrained
Δρ_max_ = 0.97 e Å^−3^
Δρ_min_ = −1.56 e Å^−3^

### 

Data collection: *CrystalClear* (Rigaku, 2005 ▶); cell refinement: *CrystalClear*; data reduction: *CrystalClear*; program(s) used to solve structure: *SHELXS97* (Sheldrick, 2008 ▶); program(s) used to refine structure: *SHELXL97* (Sheldrick, 2008 ▶); molecular graphics: *SHELXTL* (Sheldrick, 2008 ▶); software used to prepare material for publication: *SHELXTL*.

## Supplementary Material

Crystal structure: contains datablock(s) I, global. DOI: 10.1107/S1600536811055887/cv5212sup1.cif

Structure factors: contains datablock(s) I. DOI: 10.1107/S1600536811055887/cv5212Isup2.hkl

Additional supplementary materials:  crystallographic information; 3D view; checkCIF report

## Figures and Tables

**Table 1:** Hydrogen-bond geometry (Å, °)

*D*—H⋯*A*	*D*—H	H⋯*A*	*D*⋯*A*	*D*—H⋯*A*
C3—H3*C*⋯Br1	0.96	2.86	3.776 (7)	160
C2—H2*B*⋯Br2^i^	0.96	2.87	3.743 (7)	151

Symmetry code: (i) 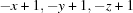.

**Fig. 1.**
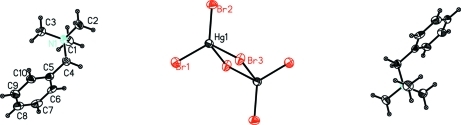
The molecular structure of the title compound showing the atomic numbering and 30% probability displacement ellipsoids [symmetry code: (A) 1-x, 1-y, 1-z]. Dashed lines denote C—H···Br interactions.

## supplementary crystallographic information

### Comment

Recently much attention has been devoted to simple molecular–ionic compounds containing organic ammonium cations and anions due to the tunability of their special structural features and their ferroelectric-dielectric properties (Nockemann & Meyer, 2002; Jin *et al.*, 2011). Herewith we present the crystal structure of the title compound.

The title compound, (C_10_H_16_N^+^)_2_^.^Hg_2_Br_6_^2-^, crystallizes in the triclinic P-1 space group (Fig. 1). The rigid [Hg_2_Br_6_]^2-^ anion situtaed on inversion center consists of two distorted tetrahedrons sharing one common edge. The terminal Hg—Br bond lengths are 2.4910 (10) and 2.4696 (8) Å, respectively, and the Br—Hg—Br bond angles are in the range 107.16 (3)° - 122.27 (3)°. The bridging Hg—Br bond lengths are 2.6039 (8) and 2.8320 (8) Å, respectively, and the bond angles of Br—Hg—Br are 102.21 (3)° - 106.41 (3)°.

In the crystal, weak intermolecular C—H···Br hydrogen bonds (Table 1) link anion and two cations into a neutral cluster (Fig. 1).

### Experimental

In room temperature benzyltrimethylammoniumchlorine (10 mmol, 1.86 g) in 20 ml water, then a water solution with HgBr_2_ (5 mmol, 1.36 g) was dropped slowly into the previous solution with properly sirring. Single crystals suitable for X-ray structure analysis were obtained by the slow evaporation of the above solution after one week in air with some colorless solid blocks appeared after days.

The dielectric constant of the compound as a function of temperature indicates that the permittivity is basically temperature-independent (ε = C/(T–T_0_)), suggesting that this compound is not ferroelectric or there may be no distinct phase transition occurring within the measured temperature (below the melting point).

### Refinement

H atoms were placed in calculated positions(C—H = 0.93Å for C*sp^2^* atoms and C—H = 0.96 Å and 0.97Å for C*sp^3^* atoms), assigned fixed *U*_iso_ values [*U*_iso_ = 1.2*U*eq(C*sp^2^*/N) and 1.5*U*eq(C*sp^3^*)] and allowed to ride.

### Crystal data

**Table:**

(C_10_H_16_N)_2_[Hg_2_Br_6_]	*Z* = 1
*M**_r_* = 1181.06	*F*(000) = 536
Triclinic, *P*1	*D*_x_ = 2.686 Mg m^−^^3^
Hall symbol: -P 1	Mo *K*α radiation, λ = 0.71073 Å
*a* = 9.0542 (11) Å	Cell parameters from 6235 reflections
*b* = 9.7287 (9) Å	θ = 6.4–26°
*c* = 9.894 (1) Å	µ = 18.72 mm^−^^1^
α = 80.78 (1)°	*T* = 298 K
β = 71.02 (1)°	Block, colourless
γ = 62.39 (1)°	0.26 × 0.22 × 0.20 mm
*V* = 730.24 (13) Å^3^	

### Data collection

**Table:**

Rigaku Mercury2 diffractometer	2872 independent reflections
Radiation source: fine-focus sealed tube	2166 reflections with *I* > 2σ(*I*)
graphite	*R*_int_ = 0.048
Detector resolution: 13.6612 pixels mm^-1^	θ_max_ = 26.0°, θ_min_ = 3.2°
CCD_Profile_fitting scans	*h* = −11→11
Absorption correction: multi-scan (*CrystalClear*; Rigaku, 2005)	*k* = −11→11
*T*_min_ = 0.011, *T*_max_ = 0.024	*l* = −12→12
6877 measured reflections	

### Refinement

**Table:**

Refinement on *F*^2^	Primary atom site location: structure-invariant direct methods
Least-squares matrix: full	Secondary atom site location: difference Fourier map
*R*[*F*^2^ > 2σ(*F*^2^)] = 0.052	Hydrogen site location: inferred from neighbouring sites
*wR*(*F*^2^) = 0.131	H-atom parameters constrained
*S* = 1.07	*w* = 1/[σ^2^(*F*_o_^2^) + (0.0524*P*)^2^ + 4.9831*P*] where *P* = (*F*_o_^2^ + 2*F*_c_^2^)/3
2862 reflections	(Δ/σ)_max_ < 0.001
139 parameters	Δρ_max_ = 0.97 e Å^−^^3^
0 restraints	Δρ_min_ = −1.56 e Å^−^^3^

### Special details

**Table:**

Geometry. All e.s.d.'s (except the e.s.d. in the dihedral angle between two l.s. planes) are estimated using the full covariance matrix. The cell e.s.d.'s are taken into account individually in the estimation of e.s.d.'s in distances, angles and torsion angles; correlations between e.s.d.'s in cell parameters are only used when they are defined by crystal symmetry. An approximate (isotropic) treatment of cell e.s.d.'s is used for estimating e.s.d.'s involving l.s. planes.
Refinement. Refinement of *F*^2^ against ALL reflections. The weighted *R*-factor *wR* and goodness of fit *S* are based on *F*^2^, conventional *R*-factors *R* are based on *F*, with *F* set to zero for negative *F*^2^. The threshold expression of *F*^2^ > σ(*F*^2^) is used only for calculating *R*-factors(gt) *etc*. and is not relevant to the choice of reflections for refinement. *R*-factors based on *F*^2^ are statistically about twice as large as those based on *F*, and *R*- factors based on ALL data will be even larger.

### Fractional atomic coordinates and isotropic or equivalent isotropic displacement parameters (Å^2^)

**Table:**

	*x*	*y*	*z*	*U*_iso_*/*U*_eq_	
Br1	0.58630 (9)	0.70394 (8)	0.72319 (8)	0.0764 (2)	
Br2	0.21880 (9)	0.50624 (8)	0.89596 (8)	0.0674 (2)	
Br3	0.30736 (8)	0.68625 (8)	0.47194 (7)	0.0655 (2)	
C1	0.9659 (8)	0.6586 (7)	0.3266 (8)	0.064 (2)	
H1A	0.9220	0.6131	0.4143	0.095*	
H1B	1.0736	0.6560	0.3256	0.095*	
H1C	0.9853	0.6010	0.2473	0.095*	
C2	0.6710 (7)	0.8258 (7)	0.3234 (7)	0.062 (2)	
H2A	0.6334	0.7749	0.4096	0.094*	
H2B	0.6846	0.7740	0.2423	0.094*	
H2C	0.5859	0.9319	0.3234	0.094*	
C3	0.8111 (8)	0.9073 (7)	0.4372 (7)	0.061 (2)	
H3A	0.7292	1.0135	0.4305	0.092*	
H3B	0.9198	0.9029	0.4358	0.092*	
H3C	0.7667	0.8621	0.5248	0.092*	
C4	0.8986 (6)	0.8952 (6)	0.1764 (6)	0.0449 (16)	
H4A	0.9305	0.8282	0.0987	0.054*	
H4B	0.8017	0.9924	0.1643	0.054*	
C5	1.0480 (6)	0.9265 (6)	0.1642 (5)	0.0413 (16)	
C6	1.2150 (8)	0.8236 (6)	0.1077 (6)	0.050 (2)	
H6	1.2390	0.7280	0.0772	0.059*	
C7	1.3495 (8)	0.8583 (8)	0.0949 (7)	0.060 (2)	
H7	1.4641	0.7856	0.0583	0.072*	
C8	1.3137 (7)	1.0004 (9)	0.1363 (6)	0.065 (2)	
H8	1.4047	1.0236	0.1292	0.078*	
C9	1.1445 (7)	1.1097 (7)	0.1885 (7)	0.058 (2)	
H9	1.1201	1.2079	0.2134	0.069*	
C10	1.0142 (7)	1.0708 (7)	0.2027 (7)	0.0511 (19)	
H10	0.8994	1.1431	0.2392	0.061*	
Hg1	0.45270 (3)	0.53868 (3)	0.69985 (3)	0.05152 (7)	
N1	0.8382 (5)	0.8208 (5)	0.3156 (5)	0.0421 (13)	

### Atomic displacement parameters (Å^2^)

**Table:**

	*U*^11^	*U*^22^	*U*^33^	*U*^12^	*U*^13^	*U*^23^
Br1	0.0961 (3)	0.0828 (3)	0.0710 (4)	−0.0584 (3)	−0.0195 (3)	−0.0032 (3)
Br2	0.0621 (3)	0.0625 (3)	0.0664 (4)	−0.0297 (2)	0.0040 (3)	−0.0121 (3)
Br3	0.0604 (3)	0.0597 (4)	0.0624 (3)	−0.0061 (3)	−0.0252 (3)	−0.0135 (3)
C1	0.064 (3)	0.052 (3)	0.074 (4)	−0.023 (3)	−0.029 (3)	0.011 (3)
C2	0.053 (2)	0.075 (3)	0.073 (4)	−0.037 (2)	−0.024 (3)	0.005 (3)
C3	0.068 (3)	0.073 (3)	0.049 (3)	−0.038 (2)	−0.005 (3)	−0.018 (3)
C4	0.058 (2)	0.042 (2)	0.048 (3)	−0.0279 (18)	−0.029 (2)	0.013 (2)
C5	0.041 (2)	0.049 (3)	0.031 (2)	−0.015 (2)	−0.0154 (19)	0.005 (2)
C6	0.065 (3)	0.034 (3)	0.031 (3)	−0.014 (2)	−0.004 (2)	0.001 (2)
C7	0.042 (3)	0.072 (4)	0.045 (3)	−0.013 (3)	−0.009 (3)	0.010 (3)
C8	0.046 (2)	0.119 (5)	0.041 (3)	−0.042 (3)	−0.020 (2)	0.011 (3)
C9	0.060 (3)	0.067 (3)	0.053 (3)	−0.037 (2)	−0.014 (3)	0.006 (3)
C10	0.038 (2)	0.053 (3)	0.056 (3)	−0.023 (2)	0.002 (2)	−0.008 (2)
Hg1	0.05128 (9)	0.05610 (11)	0.04991 (12)	−0.02925 (7)	−0.00626 (9)	−0.00723 (9)
N1	0.0451 (17)	0.041 (2)	0.048 (2)	−0.0216 (14)	−0.0197 (16)	−0.0010 (17)

### Geometric parameters (Å, °)

**Table:**

Br1—Hg1	2.4909 (10)	C4—N1	1.509 (7)
Br2—Hg1	2.4698 (8)	C4—H4A	0.9700
Br3—Hg1^i^	2.6039 (8)	C4—H4B	0.9700
Br3—Hg1	2.8318 (8)	C5—C6	1.351 (7)
C1—N1	1.475 (7)	C5—C10	1.381 (9)
C1—H1A	0.9600	C6—C7	1.371 (11)
C1—H1B	0.9600	C6—H6	0.9300
C1—H1C	0.9600	C7—C8	1.366 (11)
C2—N1	1.468 (8)	C7—H7	0.9300
C2—H2A	0.9600	C8—C9	1.377 (7)
C2—H2B	0.9600	C8—H8	0.9300
C2—H2C	0.9600	C9—C10	1.359 (10)
C3—N1	1.469 (8)	C9—H9	0.9300
C3—H3A	0.9600	C10—H10	0.9300
C3—H3B	0.9600	Hg1—Br1	2.4909 (10)
C3—H3C	0.9600	Hg1—Br3^i^	2.6039 (8)
C4—C5	1.484 (9)		
			
Hg1^i^—Br3—Hg1	91.14 (2)	C5—C6—H6	119.5
N1—C1—H1A	109.5	C7—C6—H6	119.5
N1—C1—H1B	109.5	C8—C7—C6	119.4 (5)
H1A—C1—H1B	109.5	C8—C7—H7	120.3
N1—C1—H1C	109.5	C6—C7—H7	120.3
H1A—C1—H1C	109.5	C7—C8—C9	120.8 (7)
H1B—C1—H1C	109.5	C7—C8—H8	119.6
N1—C2—H2A	109.5	C9—C8—H8	119.6
N1—C2—H2B	109.5	C10—C9—C8	118.4 (7)
H2A—C2—H2B	109.5	C10—C9—H9	120.8
N1—C2—H2C	109.5	C8—C9—H9	120.8
H2A—C2—H2C	109.5	C9—C10—C5	121.6 (5)
H2B—C2—H2C	109.5	C9—C10—H10	119.2
N1—C3—H3A	109.5	C5—C10—H10	119.2
N1—C3—H3B	109.5	Br2—Hg1—Br1	122.26 (3)
H3A—C3—H3B	109.5	Br2—Hg1—Br1	122.26 (3)
N1—C3—H3C	109.5	Br2—Hg1—Br3^i^	122.24 (3)
H3A—C3—H3C	109.5	Br1—Hg1—Br3^i^	107.18 (3)
H3B—C3—H3C	109.5	Br1—Hg1—Br3^i^	107.18 (3)
C5—C4—N1	115.1 (5)	Br2—Hg1—Br3	106.40 (3)
C5—C4—H4A	108.5	Br1—Hg1—Br3	102.23 (3)
N1—C4—H4A	108.5	Br1—Hg1—Br3	102.23 (3)
C5—C4—H4B	108.5	Br3^i^—Hg1—Br3	88.86 (2)
N1—C4—H4B	108.5	C2—N1—C3	108.1 (4)
H4A—C4—H4B	107.5	C2—N1—C1	109.7 (5)
C6—C5—C10	118.8 (6)	C3—N1—C1	108.7 (5)
C6—C5—C4	122.6 (6)	C2—N1—C4	108.5 (5)
C10—C5—C4	118.4 (4)	C3—N1—C4	110.4 (5)
C5—C6—C7	121.0 (6)	C1—N1—C4	111.4 (4)
			
N1—C4—C5—C6	91.4 (6)	C6—C5—C10—C9	−1.7 (9)
N1—C4—C5—C10	−93.3 (6)	C4—C5—C10—C9	−177.2 (6)
C10—C5—C6—C7	3.1 (9)	Hg1^i^—Br3—Hg1—Br2	−123.39 (3)
C4—C5—C6—C7	178.3 (5)	Hg1^i^—Br3—Hg1—Br1	107.33 (3)
C5—C6—C7—C8	−1.8 (9)	Hg1^i^—Br3—Hg1—Br1	107.33 (3)
C6—C7—C8—C9	−1.1 (10)	C5—C4—N1—C2	168.8 (4)
C7—C8—C9—C10	2.4 (10)	C5—C4—N1—C3	50.6 (5)
C8—C9—C10—C5	−1.0 (10)	C5—C4—N1—C1	−70.3 (7)

Symmetry codes: (i) −*x*+1, −*y*+1, −*z*+1.

### Hydrogen-bond geometry (Å, °)

**Table:**

*D*—H···*A*	*D*—H	H···*A*	*D*···*A*	*D*—H···*A*
C3—H3C···Br1	0.96	2.86	3.776 (7)	160
C2—H2B···Br2^i^	0.96	2.87	3.743 (7)	151

Symmetry codes: (i) −*x*+1, −*y*+1, −*z*+1.

## References

[bb1] Jin, L. & Liu, N. (2011). *Acta Cryst.* E**67**, m1586. 10.1107/S1600536811043091 PMC3246999 22219819

[bb2] Nockemann, P. & Meyer, G. (2002). *Acta Cryst.* E**58**, m529–m530.

[bb3] Rigaku (2005). *CrystalClear* Rigaku Corporation, Tokyo, Japan.

[bb4] Sheldrick, G. M. (2008). *Acta Cryst.* A**64**, 112–122. 10.1107/S0108767307043930 18156677

